# Integrated DNA Methylation and Transcriptome Analysis Reveals Epigenetic Mechanisms of Lactation Performance Differences in Cloned Buffalo

**DOI:** 10.3390/ijms262311585

**Published:** 2025-11-29

**Authors:** Jia-Hao Hu, Hai-Ying Zheng, Chun-Yan Yang, Jiang-Hua Shang

**Affiliations:** 1 Guangxi Key Laboratory of Buffalo Genetics, Breeding and Reproduction, Guangxi Buffalo Research Institute, Chinese Academy of Agricultural Sciences, Nanning 530001, China; hjh202510@126.com (J.-H.H.); haiyingzheng@126.com (H.-Y.Z.); 2 Key Laboratory of Buffalo Genetics, Breeding and Reproduction Technology, Ministry of Agriculture and Rural Affairs, Nanning 530001, China

**Keywords:** cloned buffalo, milk yield variation, DNA methylation, transcriptome, epigenetic regulation

## Abstract

Buffalo milk plays a vital role in the dairy industry, with milk yield regulated by both transcriptomic and epigenetic mechanisms. While previous studies have primarily focused on differences among individuals or breeds, the epigenetic basis underlying milk yield variation in genetically identical animals remains poorly understood. In this study, we employed a cloned buffalo model and integrated whole-genome bisulfite sequencing (WGBS) with RNA sequencing (RNA-seq) to investigate how DNA methylation and transcriptional regulation contribute to milk yield variation. Results tentatively revealed that low-yielding buffalo exhibited globally reduced DNA methylation in mammary tissues, with distinct distribution patterns across genomic features and regulatory regions. Differentially methylated genes were enriched in PI3K-Akt, HIF-1, and immune-related pathways, whereas hypomethylated genes were associated with calcium signaling, cAMP pathways, and metabolic processes. Transcriptome analysis showed that high-yielding buffalo upregulated genes involved in lipid metabolism and cell proliferation, while low-yielding buffalo displayed enrichment in immune stress and amino acid metabolism. Integrative analysis identified 126 hypo-upregulated genes and highlighted hub regulators such as KLF6, NR4A1, ESR1, KCNQ1. Collectively, this study outlines a preliminary multi-omics regulatory landscape of milk yield variation in cloned buffalo, suggests the interplay between DNA methylation and transcription, provides preliminary insights into the potential interplay between DNA methylation and transcription, and suggests potential connections that merit further investigation.

## 1. Introduction

Buffalo (*Bubalus bubalis*), a vital livestock species in tropical and subtropical regions, plays a crucial role in dairy production [1]. Buffalo milk is distinguished by its high nutritional value. In comparison with cow milk, it exhibits a higher fat content (8.0%), an increased proportion of unsaturated fatty acids, elevated protein levels (4.5%), along with relatively lower phospholipid and cholesterol concentrations [2,3]. Despite these advantages, significant variations in milk yield among individuals remain a major constraint on buffalo dairy production. Therefore, understanding the molecular and epigenetic mechanisms underlying this variability is crucial for improving lactation performance and breeding efficiency.

The rapid advancement of omics technologies has provided powerful tools for revealing the molecular mechanisms underlying lactation traits [4,5]. Transcriptome analyses have identified differentially expressed genes (DEGs) in the mammary tissues of buffaloes and bovines with high and low yields [6,7]. These genes are predominantly enriched in pathways associated with milk protein and fat metabolism, mammary gland development, and immune responses. Based on a transcriptomic study of Dehong buffalo, in the comparison between high and low-fat groups, key genes in milk fat synthesis along with genes related to immune functions were found to be differentially expressed [8]. In cattle research, transcriptomic comparisons of high- and low-yield groups showed that DEGs were concentrated in Toll-like receptor signaling, inflammatory responses, and fatty acid metabolism pathways, highlighting the significance of immune and metabolic processes in milk production regulation [9,10].

Epigenetic modifications, especially DNA methylation, have been shown to be involved in lactation regulation. Dysregulation of these modifications can significantly impact milk synthesis [4]. In the case of goats, a high-concentrate diet suppresses the expression of specific genes by increasing the methylation level in their promoter regions, thereby altering milk fat composition and causing a decrease in milk fat percentage [11]. Similarly, investigations into bovine mammary epithelial cells have disclosed that genetic variation influencing lipid metabolism and immune pathways is intricately linked to disparities in lactation performance [12]. Nevertheless, most of these studies have focused on inter-individual variation caused by genetic background or environmental conditions, while the contribution of epigenetic differences among genetically identical individuals to milk yield regulation remains insufficiently explored.

Somatic cell nuclear transfer (SCNT)-derived cloned animals provide an ideal model for investigating epigenetic regulatory mechanisms that underlie phenotypic variation among genetically identical individuals [13,14]. Given that cloned individuals share identical nuclear genomes yet often display variable epigenetic reprogramming, they represent a powerful system for isolating non-genetic sources of phenotypic variation. Previous studies have reported that SCNT cattle often exhibit lower protein and fat yields during the first lactation compared with non-cloned counterparts, indicating phenotypic variability in milk composition and metabolism [15]. Such variation is commonly ascribed to incomplete epigenetic reprogramming during embryonic and early developmental stages, including delayed or abnormal erasure and re-establishment of DNA methylation, along with impaired activation of imprinted or developmentally essential genes [13,14,16]. Despite numerous studies on animals with diverse genetic backgrounds or environments, understanding the sole contribution of epigenetic variation to lactation performance remains a gap. Cloned animals via SCNT, having an identical nuclear genome but distinct phenotypic differences, such as in milk yield, present a unique opportunity to bridge this gap.

Consequently, mammary gland tissues sourced from two cloned buffaloes were procured and comprehensively analyzed via transcriptome sequencing and whole-genome bisulfite sequencing for DNA methylation profiling. In this study, we aim to identify key genes and methylation regions associated with lactation disparities, thus uncovering the underlying mechanisms through which epigenetic modifications regulate milk yield.

## 2. Result

### 2.1. Genome-Wide DNA Methylation Landscapes in Mammary Tissues of Cloned Buffalo

**Figure 1:**
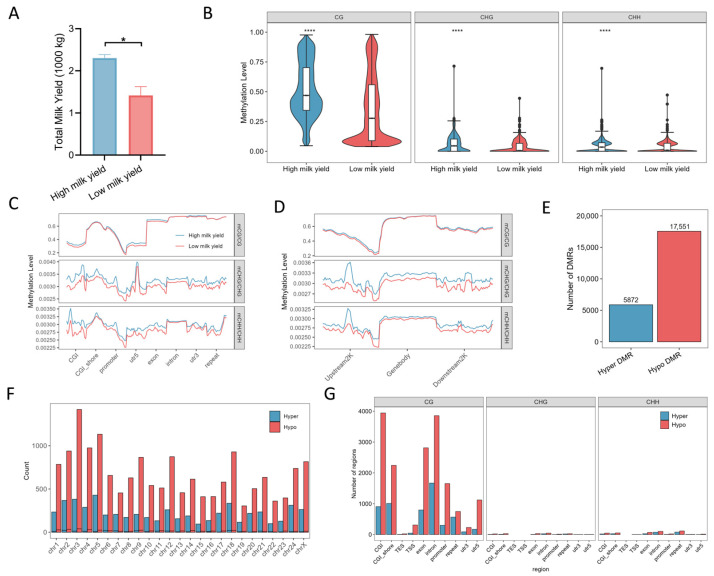
Global DNA methylation profiles in cloned buffalo (**A**) Milk yield differences between the high milk yield and low milk yield buffalo (*n* = 3, *p* = 0.0166, SD ± SEM). (**B**) Comparison of global methylation levels across CG, CHG, and CHH contexts between the high milk yield and low milk yield buffalo. (**C**,**D**). Methylation distribution patterns in genomic features such as CpG islands, exons, and introns. (**E**) Numbers of hypermethylated differentially methylated regions (DMRs) (regions with ≥10% higher methylation relative to the other buffalo) and hypomethylated DMRs (regions with ≥10% lower methylation relative to the other buffalo). (**F**) Chromosomal distribution of DMRs. (**G**) Annotation of DMRs across genomic features across CG, CHG, and CHH contexts. (* represent *p* value < 0.05, **** represent *p* value <0.0001).

We performed whole-genome bisulfite sequencing (WGBS) on mammary tissues from two cloned buffalo. Total milk yields were recorded in three consecutive measurements; the low-yield individual produced significantly less milk than the high-yield counterpart (Figure 1A). Methyl sequencing and data analysis were performed. The results showed that an average of 188,601,081 raw reads were obtained for each sample. After filtering, the clean reads (Table S1) were compared to the reference genome. The proportion of clean reads that could be mapped to a unique position of the reference sequence ranged from 80 to 82%.

Global methylation profiling revealed that methylation levels in the CG, CHG and CHH contexts were markedly reduced in the low-yield buffalo compared with the high-yield buffalo (Figure 1B). To examine locus-specific patterns, we further analyzed methylation across genomic functional elements and flanking regions. In the CG context, methylation within CpG islands (CGI), 5′ UTRs and exons were lower in the low-yield individual than in the high-yield individual, while in CHG/CHH contexts methylation was reduced across all annotated genomic elements in the low-yield sample (Figure 1C). Analysis of upstream/downstream regions indicated that, in the CG context, methylation in Upstream2K and Downstream2K was slightly higher in the low-yield animal; conversely, in CHG and CHH contexts methylation across gene bodies, Upstream2K and Downstream2K were higher in the low-yield sample (Figure 1D).

Differential methylation analysis using the DSS R package identified differentially methylated regions (DMRs). For this study, DMRs were identified as regions showing ≥10% methylation difference between the high- and low-yield buffalo. After filtering, we detected 5872 hypermethylated DMRs (Hyper-DMRs) and 17,551 hypomethylated DMRs (Hypo-DMRs) (Figure 1E). Chromosomal distribution analysis indicated that hypomethylated DMRs showed a more clustered distribution across specific chromosomes (Figure 1F). Further analysis of the regions of DMRs revealed that DMRs were more abundant in the CG context and were concentrated in the intron, CGI, and exon regions, while being less prevalent in the CHG and CHH contexts (Figure 1G).

### 2.2. Functional Annotation and Pathway Enrichment of Differentially Methylated Genes

**Figure 2:**
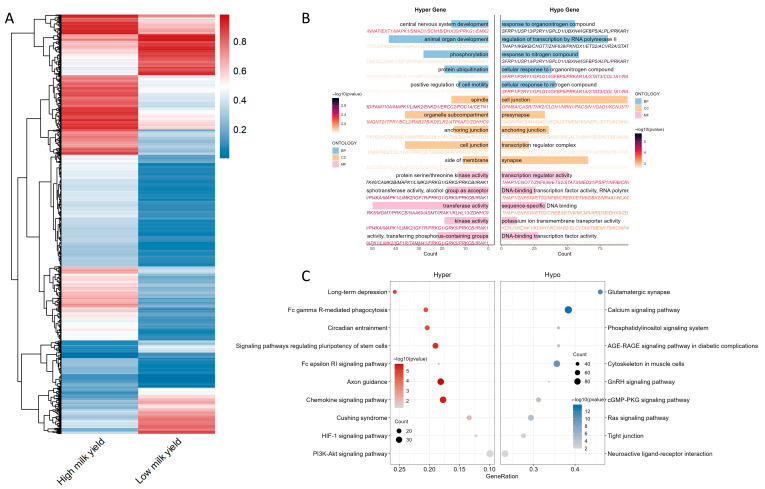
Functional annotation of differentially methylated genes. (**A**) Heatmap of methylation levels of differentially methylated genes (DMGs) across samples. (**B**) GO enrichment results comparing hypo- and hypermethylated DMGs. (**C**) KEGG pathway enrichment bubble plots of hypo- and hypermethylated DMGs.

To characterize differentially methylated genes (DMGs), we annotated DMRs against the reference genome GCF_019923935.1 and defined genes as DMGs when the associated DMRs showed an absolute methylation difference greater than 0.1 between the two buffalo. Based on this comparative definition, we identified 4750 hypomethylated genes and 1910 hypermethylated genes (Figure 2A). Functional enrichment analyses (GO and KEGG) showed that hypermethylated genes were predominantly associated with processes such as central nervous system development, protein ubiquitination, positive regulation of cell migration, anchoring junctions and centromere/kinetochore structures. KEGG enrichment implicated axon guidance, FcεRI signaling, PI3K-Akt, Cushing syndrome, HIF-1 signaling and FcγR-mediated phagocytosis, suggesting that hypermethylation may repress pathways relevant to secretory activity in mammary epithelial cells. In contrast, hypomethylated genes were enriched in GO terms related to organic nitrogen response, transcriptional regulation, synaptic architecture and transcription factor activity, and in KEGG pathways including calcium signaling, glutamatergic synapse, muscle cytoskeleton, cGMP-PKG signaling, neuroactive ligand-receptor interaction, phosphatidylinositol signaling, Ras signaling, GnRH signaling and AGE-RAGE signaling (Figure 2B,C), processes closely tied to secretion, milk biosynthesis and energy metabolism in mammary epithelium.

### 2.3. Transcriptomic Profiling Reveals Differential Gene Expression Associated with Milk Yield

**Figure 3:**
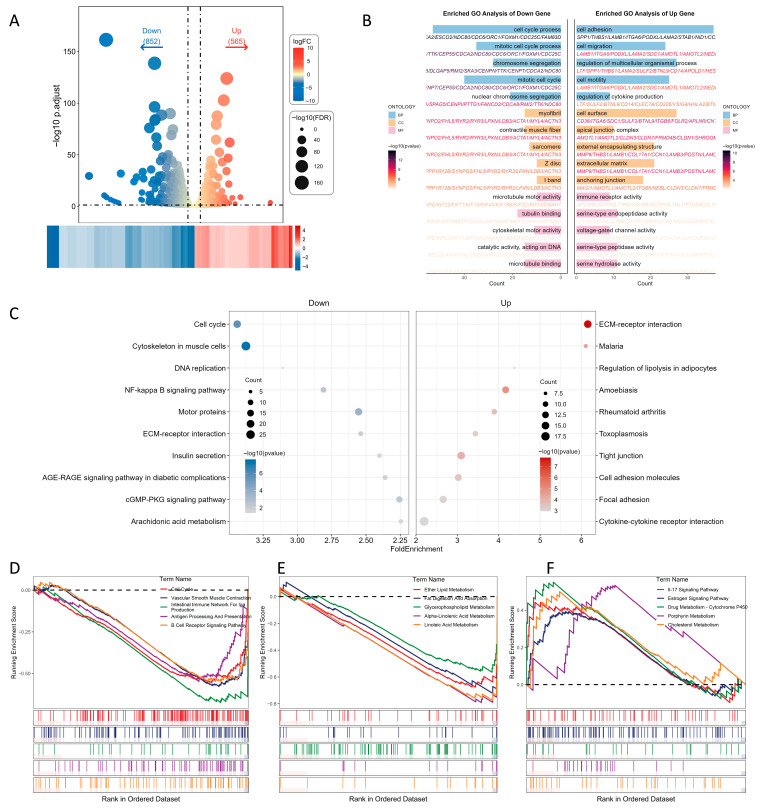
Transcriptome characteristics in mammary tissue of cloned buffalo. (**A**) Volcano plot and heatmap showing differentially expressed genes (DEGs) between high- and low-yield groups. (**B**) GO enrichment analysis of upregulated and downregulated genes. (**C**) KEGG pathway enrichment analysis of upregulated and downregulated genes. (**D**–**F**) GSEA-KEGG enrichment results for representative pathways (*p* < 0.05).

For transcriptomic profiling, we performed differential expression analysis with the edgeR package. In the low-yield buffalo, 565 genes were identified as upregulated and 852 genes as downregulated; a heatmap of logFC values depicts these expression changes (Figure 3A).

GO analysis indicated that the upregulated genes were mainly involved in responses to lipids and oxygen-containing compounds, organ development and regulation of cell proliferation, and localized to the cell membrane and apical plasma membrane, with roles in signal transduction and receptor binding. Downregulated genes were primarily associated with cell division, chromosome segregation and muscle development and were enriched for functions related to myofibrils, synapses and potassium channel activity (Figure 3B).

KEGG analysis showed that upregulated genes were enriched in pathways related to ECM–receptor interaction, tight junctions and phagosome function, whereas downregulated genes were enriched in cell cycle, cytoskeletal regulation and cell adhesion processes (Figure 3C). GSEA further indicated that high-yield buffalo are characterized by enrichment in cell cycle and lipid metabolism pathways, together with more stable immune homeostasis and improved mammary blood supply (Figure 3D,E). By contrast, the low-yield animal showed relative enrichment of pathways involved in amino acid (e.g., phenylalanine) and steroid/cholesterol metabolism, estrogen signaling and IL-17 signaling (Figure 3F), suggesting that immune, metabolic and hormonal dysregulation may contribute to reduced milk yield.

### 2.4. Integrated Methylome–Transcriptome Analysis Identifies Hypo-Up Genes and Hub Regulators

**Figure 4:**
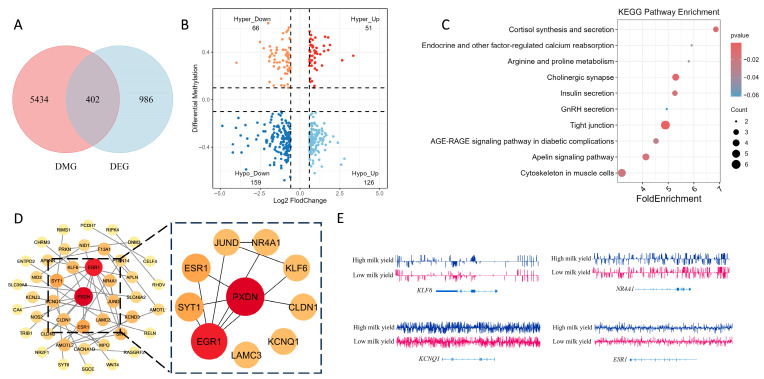
Integrated methylome-transcriptome analysis. (**A**) Venn diagram of overlap between DMGs and DEGs. (**B**) Nine-quadrant analysis of the relationship between DMGs and DEGs. (**C**) KEGG pathway enrichment analysis of hypo-up genes. (**D**) Protein–protein interaction (PPI) network of hypo-up genes and candidate hub genes. (**E**) IGV visualization of KLF6, NR4A1, ESR1, and KCNQ1 genes showing methylation differences between high- and low-yield groups.

To identify expression changes associated with differential methylation, we integrated methylome and transcriptome data. A Venn analysis showed an overlap of 493 genes between differentially methylated genes (DMGs) and differentially expressed genes (DEGs) (Figure 4A). DNA methylation levels at individual genomic features exhibited an overall inverse relationship with gene expression. Nine-quadrant analysis of gene logFC and differential methylation identified 126 hypo-up genes (hypomethylated with increased expression) (Figure 4B).

KEGG enrichment of these genes implicated insulin secretion, cAMP and calcium signaling, tight junctions, ECM-receptor interaction, Apelin signaling, AGE-RAGE signaling and various amino acid metabolism pathways, indicating roles in hormonal regulation, metabolic signaling and maintenance of mammary structure. Protein–protein interaction (PPI) network analysis further identified several candidate hub genes (Figure 4C). Comparison with the Animal QTLdb for cattle showed that KLF6 maps to QTL regions for milk yield, milk fat and milk protein traits, while NR4A1, ESR1 and KCNQ1 also co-localize with QTLs linked to lactation performance and udder conformation. IGV visualization of these representative hub genes demonstrated distinct methylation differences between high- and low-yield buffalo samples (Figure 4D).

## 3. Discussion

Milk yield is recognized as one of the most important economic traits in buffalo, exerting a direct influence on the profitability of the dairy industry. The molecular mechanisms underlying variation in milk production have been increasingly investigated, with epigenetic modifications—particularly DNA methylation—being highlighted for their roles in mammary epithelial cell development, energy metabolism, and secretory regulation [17,18,19]. Despite an identical genetic background, significant differences in milk yield have been observed among cloned individuals, indicating that non-genetic factors, especially epigenetic regulation, may play a pivotal role in shaping lactation performance. In the present study, a cloned buffalo model was employed, and whole-genome bisulfite sequencing (WGBS) together with RNA sequencing (RNA-seq) were integrated to systematically explore the contributions of DNA methylation and transcriptional regulation to high- and low-yield phenotypes.

DNA methylation is considered a core epigenetic mechanism regulating mammary gland development and lactation performance, and its aberrations have frequently been linked to milk yield variation [4,19]. In cloned buffalo, dysregulation of this mechanism is thought to amplify reprogramming defects inherent to somatic cell nuclear transfer, thereby contributing to phenotypic divergence among genetically identical individuals. Recent genomic analyses of SCNT-derived buffalo clones have similarly reported detectable methylation variability despite near-identical nuclear genomes, supporting the notion that epigenetic divergence can arise within cloned individuals [20]. In this study, a global hypomethylation pattern was revealed in the mammary tissues of low-yield buffalo by WGBS, which is likely to influence the transcriptional activity of lactation-related genes through selective modifications in promoter and gene body regions. This pattern resembled methylation changes reported in nutritional stress models of cattle [5,21]. Specifically, reduced methylation in CGI and exon regions under the CG context was associated with potential activation of transcription initiation, whereas global decreases in CHG/CHH contexts may facilitate the activation of stress-response genes, leading to energy allocation toward defense rather than milk synthesis. Such context-dependent regulation suggests metabolic vulnerability in low-yield buffalo, consistent with inflammation cascades induced by methylation dysregulation in bovine mastitis [19]. Furthermore, the distribution of differentially methylated regions (DMRs) supported this interpretation, as hypomethylated regions concentrated in intron, CGI, and exon regions may constrain mammary epithelial adaptability, resembling the role of methylation patterns in determining tissue specificity observed in duck muscle development [22].

Functional annotation of differentially methylated genes further revealed that hypermethylated genes were enriched in pathways including axon guidance, chemokine signaling, pluripotency stem cell signaling, long-term depression, circadian rhythm, FcγR-mediated phagocytosis, Fc epsilon RI signaling, PI3K-Akt signaling, Cushing’s syndrome, and HIF-1 signaling. The combined suppression of these pathways is likely to impair mammary epithelial cell proliferation, hormone responsiveness, and stress adaptation, ultimately leading to reduced milk yield. Notably, inhibition of the PI3K-Akt pathway has been associated with decreased milk protein synthesis in cattle [23], while dysregulation of the HIF-1 pathway has been suggested to hinder mammary adaptation to hypoxic environments, thereby affecting energy allocation [24]. In contrast, hypomethylated genes (high transcription) were enriched in calcium signaling, glutamatergic synapse, muscle cell cytoskeleton, cGMP-PKG signaling, neuroactive ligand-receptor interaction, phosphatidylinositol signaling, Ras signaling, and GnRH signaling. Activation of these pathways may induce metabolic disturbances and stress cascades, indirectly suppressing milk synthesis. In bovine models, excessive activation of Ras and JAK-STAT has been linked to fatty acid-induced impairment of milk protein and fat synthesis, while dysregulation of calcium signaling and cGMP-PKG has been reported to disrupt calcium homeostasis in the mammary gland, leading to reduced secretory efficiency [22,25].

Transcriptomic differences further reinforced this mechanism. Downregulation of genes related to cell cycle, cytoskeletal organization and cell adhesion in the low-yield group was suggestive of restricted renewal of mammary epithelial cells, in stark contrast to the activation of the ECM–receptor interaction, tight junction and phagosome pathways observed in the high-yield group, which may promote epithelial function. Similar patterns have been reported in bovine transcriptome studies, where cell cycle activation was positively associated with milk production [25]. GSEA results indicated that pathways such as cell cycle, vascular smooth muscle contraction, and glycerophospholipid metabolism were activated in the high-yield group, thereby regulating mammary cell division, nutrient transport, and lipid biosynthesis to directly enhance milk yield [26,27]. The JAK-STAT signaling pathway was also activated in high-yield individuals, likely promoting milk protein expression through prolactin and other hormone responses [28]. In contrast, activation of the IL-17 signaling pathway in the low-yield group was observed, which may trigger inflammatory cascades, disrupt mammary homeostasis, and consequently reduce production [29,30]. Collectively, these results indicate that transcriptomic differences influence buffalo lactation performance by regulating key signaling networks, with epigenetic variation in cloned animals potentially amplifying transcriptional responses and contributing to phenotypic inconsistency.

Differences in milk yield are essentially driven by regulatory variations in mammary epithelial cells involving energy metabolism, secretory activity, and tissue structural maintenance. Previous studies have demonstrated that key lactation-related signals include insulin and hormonal regulation, calcium homeostasis, cell–matrix interactions, and the permeability of tight junctions, all of which collectively determine the efficiency of milk synthesis and secretion [31,32]. In this study, hypo-up regulated genes were enriched in multiple pathways, including insulin secretion, cAMP and calcium signaling, tight junctions, ECM-receptor interaction, Apelin signaling, AGE-RAGE signaling, and amino acid metabolism, indicating systematic alterations in secretory and metabolic networks. These hypo-up patterns suggest that methylation status and transcriptional activity shift in a coordinated manner within these pathways. The regulatory relationships underlying these associations remain unclear and will require further investigation.

A more comprehensive biological context was obtained by examining whether these hub genes have been previously reported within cattle QTL regions related to milk production and udder traits [33]. Notably, KLF6 and NR4A1 fall within QTL intervals associated with milk fat percentage, fat yield, protein percentage, protein yield, and total milk yield, whereas ESR1 and KCNQ1 are located in QTL regions linked to udder attachment and depth. KLF6 has been shown to play a critical role in activating the peroxisome proliferator-activated receptor alpha (PPARα) signaling pathway and has been identified as a potential candidate gene for milk production traits [34]. Evidence from mammary epithelial studies further indicates that KLF6 contributes to cell differentiation and lipid metabolic regulation [35]. ESR1, as a growth-related transcription factor, has been reported to drive cell proliferation and migration in mammary epithelial cells [36]. It also plays an essential regulatory role in hormone-dependent mammary gland development [37]. NR4A1 was identified as a key differential gene in a study of high- and low-yielding cattle, with significant associations observed for milk fat, milk protein, and milk yield [38]. Functional evidence shows that NR4A1 modulates metabolic and inflammatory signaling in mammary epithelial cells, indicating its relevance to lactation physiology [39]. KCNQ1, a voltage-dependent K(+) channel that regulates gastric acid secretion, salt balance, and glucose homeostasis, was strongly associated with genetic variation in udder morphology in a bovine GWAS analysis [40]. Beyond this genetic evidence, KCNQ1 also supports epithelial ion transport and secretory stability, processes fundamental to mammary epithelial function [41]. Their recurrence across genetic (QTL) and epigenetic/transcriptomic datasets suggests functional relevance in lactation biology, although these layers reflect distinct regulatory mechanisms. Taken together, hypo-upregulated genes were primarily concentrated in hormone signaling, calcium homeostasis, energy metabolism, and structural maintenance pathways, and their combined effects are likely to weaken mammary epithelial secretory capacity and metabolic efficiency, ultimately contributing to reduced lactation performance.

In summary, by integrating whole-genome methylome and transcriptome data, this study systematically revealed epigenetic and transcriptional differences between high- and low-yield cloned buffalo, providing insights into the complex interplay between DNA methylation and gene expression. A series of key pathways associated with energy metabolism, calcium signaling, and ECM-receptor interactions were identified, along with potential core regulatory genes such as KLF6, EGR1, and NR4A1, which may serve as potential candidate biomarkers for mammary function and milk yield regulation. Although the sample size of this study was limited and dynamic disease processes were not captured, the findings nonetheless provide a novel preliminary explanatory framework for differences in mammary secretion mechanisms and lactation efficiency. The major strength of this study lies in the use of a cloned buffalo model, which eliminates confounding effects of the genetic background and allows for a more precise delineation of the role of DNA methylation in lactation regulation. These discoveries not only deepen the understanding of the molecular mechanisms underlying milk yield variation but also provide preliminary theoretical foundations and potential applications for genetic improvement and sustainable dairy production.

## 4. Materials and Methods

### 4.1. Tissue Sampling

Two female cloned buffalo generated by somatic cell nuclear transfer (from the same ear fibroblast donor) were maintained at the Guangxi buffalo breeding farm under free-range conditions. Based on total milk yield and mammary gland condition, the animals were assigned to high-yield and low-yield groups. Mammary gland tissue samples were collected and immediately snap-frozen in liquid nitrogen for subsequent RNA and DNA extraction.

### 4.2. DNA and RNA Extraction

Collected mammary tissues were stored immediately in liquid nitrogen. Total RNA was extracted using TRIzol reagent (Invitrogen, Carlsbad, CA, USA), and RNA purity was assessed with a NanoDrop ND2000 spectrophotometer (Thermo Scientific, Wilmington, DE, USA). Genomic DNA was extracted from tissue samples using a DNA extraction kit (Takara, Dalian, China), and DNA purity was measured by NanoDrop ND2000. DNA integrity and potential contamination by RNA or protein were evaluated by electrophoresis on a 1% agarose gel.

### 4.3. RNA-Sequencing

High-quality RNA was used for library preparation. Qualified libraries were pooled according to effective concentration and the target sequencing depth. Base calling of sequencing images was performed using CASAVA to generate raw reads. Raw reads were trimmed with fastp to remove adaptor sequences, poly-N reads and low-quality reads, yielding clean reads. Paired-end clean reads were aligned to the buffalo reference genome (GCF_019923935.1) using HISAT2 v2.0.5 to obtain raw gene counts. Read counts were quantified with FeatureCounts and converted to transcripts per kilobase million (TPM) using in-house scripts.

Differential expression analysis was conducted using the edgeR R package (v4.0.16) [42]. A DGEList object was constructed from the count matrix, after which genes with counts per million (CPM) > 1 in at least one sample were filtered. Normalization was carried out via the trimmed mean of M-values (TMM) method, and the dispersion was estimated with a biological coefficient of variation (BCV) of 0.05. DEGs were identified based on a FDR < 0.05 and |log2 foldchange| ≥ 0.585.

### 4.4. Whole-Genome Bisulfite Sequencing

100 ng of genomic DNA were mixed with 0.5 μg unmethylated lambda DNA and sheared to 200–400 bp fragments using a Covaris S220 ultrasonicator. Bisulfite conversion of unmethylated cytosines to uracils was performed using the EZ DNA Methylation-Gold™ Kit (Zymo Research, Irvine, CA, USA), followed by small-fragment library construction. Qualified libraries were sequenced on an Illumina NovaSeq platform (Illumina, San Diego, CA, USA) using paired-end runs. Raw reads were filtered with fastp, and clean reads were aligned to the reference genome using Bismark. Reads aligning to identical genomic coordinates were considered duplicates and were removed. The results of methylation extractor were transformed into bigWig format for visualization using IGV browse (v 2.19.1). Methylated cytosines were extracted from alignments using the Bismark methylation extractor with default parameters. For each identified CpG site, the methylation level (ML) was calculated as follows.

$$ML\left(C\right)=\frac{reads(mC)}{reads\left(mC\right)+reads(C)}$$

Differentially methylated regions (DMRs) were identified using DSS (v2.50.1) [43]. DMRs were annotated according to their genomic locations and were classified as gene body regions (from TSS to TES) or promoter regions (2 kb upstream of TSS).

### 4.5. GO and KEGG Enrichment Analysis of DEGs and DMGs

GO and KEGG enrichment analyses for differentially DEGs and DMGs were performed and visualized using the clusterProfiler (v4.16.0) and ggplot2 (v3.5.2) R packages (39529960). Gene set enrichment analysis (GSEA) of RNA gene sets was visualized using the clusterProfiler and GseaVis (v0.1.1) R packages.

### 4.6. Protein–Protein Interaction

Differential gene lists were submitted to the STRING database (https://cn.string-db.org/, accessed on 22 November 2025) to retrieve predicted protein–protein interaction (PPI) information. PPI networks were imported into Cytoscape (v3.10.2) for visualization and analysis, and hub genes were identified using the cytoHubba plugin.

## Data Availability

The following data of whole-genome DNA methylation and transcriptome data of buffalo are submitted in the NCBI repository with BioProject ID PRJNA1347555. The data presented in this study are openly available.

## Supplementary Materials

The supporting information can be downloaded at: https://www.mdpi.com/article/10.3390/ijms262311585/s1.

## References

[1] Zhang Y., Colli L., Barker J.S.F. (2020). Asian water buffalo: Domestication, history and genetics. Anim. Genet..

[2] Zicarelli L. (2004). Buffalo milk: Its properties, dairy yield and mozzarella production. Vet. Res. Commun..

[3] Du C., Deng T., Zhou Y., Ye T., Zhou Z., Zhang S., Shao B., Wei P., Sun H., Khan F.A. (2019). Systematic analyses for candidate genes of milk production traits in water buffalo (*Bubalus bubalis*). Anim. Genet..

[4] Xue Q., Huang Y., Cheng C., Wang Y., Liao F., Duan Q., Wang X., Miao C. (2023). Progress in epigenetic regulation of milk synthesis, with particular emphasis on mRNA regulation and DNA methylation. Cell Cycle.

[5] Ju Z., Jiang Q., Wang J., Wang X., Yang C., Sun Y., Zhang Y., Wang C., Gao Y., Wei X. (2020). Genome-wide methylation and transcriptome of blood neutrophils reveal the roles of DNA methylation in affecting transcription of protein-coding genes and miRNAs in *E. coli*-infected mastitis cows. BMC Genom..

[6] Deng T., Liang A., Liang S., Ma X., Lu X., Duan A., Pang C., Hua G., Liu S., Campanile G. (2019). Integrative Analysis of Transcriptome and GWAS Data to Identify the Hub Genes Associated with Milk Yield Trait in Buffalo. Front. Genet..

[7] Du C., Zhu La A.T., Gao S., Gao W., Ma L., Bu D., Zhang W. (2024). Hepatic Transcriptome Reveals Potential Key Genes Contributing to Differential Milk Production. Genes.

[8] Hao M., Jiang J., Zhang Y., Wang S., Fu G., Zou F., Xie Y., Zhao S., Li W. (2021). Transcriptional profiling of buffalo mammary gland with different milk fat contents. Gene.

[9] Bai X., Zheng Z., Liu B., Ji X., Bai Y., Zhang W. (2016). Whole blood transcriptional profiling comparison between different milk yield of Chinese Holstein cows using RNA-seq data. BMC Genom..

[10] Cui X., Hou Y., Yang S., Xie Y., Zhang S., Zhang Y., Zhang Q., Lu X., Liu G.E., Sun D. (2014). Transcriptional profiling of mammary gland in Holstein cows with extremely different milk protein and fat percentage using RNA sequencing. BMC Genom..

[11] Tian P., Luo Y., Li X., Tian J., Tao S., Hua C., Geng Y., Ni Y., Zhao R. (2017). Negative effects of long-term feeding of high-grain diets to lactating goats on milk fat production and composition by regulating gene expression and DNA methylation in the mammary gland. J. Anim. Sci. Biotechnol..

[12] Ahmad S.M., Bhat B., Bhat S.A., Yaseen M., Mir S., Raza M., Iquebal M.A., Shah R.A., Ganai N.A. (2021). SNPs in Mammary Gland Epithelial Cells Unraveling Potential Difference in Milk Production Between Jersey and Kashmiri Cattle Using RNA Sequencing. Front. Genet..

[13] Matoba S., Zhang Y. (2018). Somatic Cell Nuclear Transfer Reprogramming: Mechanisms and Applications. Cell Stem Cell.

[14] Cao P., Li H., Zuo Y., Nashun B. (2020). Characterization of DNA Methylation Patterns and Mining of Epigenetic Markers During Genomic Reprogramming in SCNT Embryos. Front. Cell Dev. Biol..

[15] Montazer-Torbati F., Boutinaud M., Brun N., Richard C., Neveu A., Jaffrezic F., Laloe D., LeBourhis D., Nguyen M., Chadi S. (2016). Differences during the first lactation between cows cloned by somatic cell nuclear transfer and noncloned cows. J. Dairy Sci..

[16] Matoba S., Wang H., Jiang L., Lu F., Iwabuchi K.A., Wu X., Inoue K., Yang L., Press W., Lee J.T. (2018). Loss of H3K27me3 Imprinting in Somatic Cell Nuclear Transfer Embryos Disrupts Post-Implantation Development. Cell Stem Cell.

[17] Wijenayake S., Eisha S., Purohit M.K., McGowan P.O. (2024). Milk derived extracellular vesicle uptake in human microglia regulates the DNA methylation machinery: Short title: Milk-derived extracellular vesicles and the epigenetic machinery. Sci. Rep..

[18] Dechow C.D., Liu W.S. (2018). DNA methylation patterns in peripheral blood mononuclear cells from Holstein cattle with variable milk yield. BMC Genom..

[19] Nayan V., Singh K., Iquebal M.A., Jaiswal S., Bhardwaj A., Singh C., Bhatia T., Kumar S., Singh R., Swaroop M.N. (2022). Genome-Wide DNA Methylation and Its Effect on Gene Expression During Subclinical Mastitis in Water Buffalo. Front. Genet..

[20] Punetha M., Kumar D., Kumar S., Maggo B., Dahiya P., Kumar P., Sharma R.K., Pal Y., Yadav P.S. (2025). Unravelling High Nuclear Genomic Similarity and Mitochondria Linked Epigenetic Divergence in SCNT Derived Buffalo Clones via Long-Read Nanopore Genome Sequencing. Int. J. Mol. Sci..

[21] Wang M., Bissonnette N., Dudemaine P.L., Zhao X., Ibeagha-Awemu E.M. (2021). Whole Genome DNA Methylation Variations in Mammary Gland Tissues from Holstein Cattle Producing Milk with Various Fat and Protein Contents. Genes.

[22] Wang M., Liang Y., Ibeagha-Awemu E.M., Li M., Zhang H., Chen Z., Sun Y., Karrow N.A., Yang Z., Mao Y. (2020). Genome-Wide DNA Methylation Analysis of Mammary Gland Tissues from Chinese Holstein Cows with Staphylococcus aureus Induced Mastitis. Front. Genet..

[23] Gao Y., Liu G.E., Ma L., Fang L., Li C.J., Baldwin R.L.T. (2025). Transcriptomic profiling of gastrointestinal tracts in dairy cattle during lactation reveals molecular adaptations for milk synthesis. J. Adv. Res..

[24] Sengupta T., Abraham G., Xu Y., Clurman B.E., Minella A.C. (2011). Hypoxia-inducible factor 1 is activated by dysregulated cyclin E during mammary epithelial morphogenesis. Mol. Cell Biol..

[25] Xu Q., Li X., Ma L., Loor J.J., Coleman D.N., Jia H., Liu G., Xu C., Wang Y., Li X. (2019). Adipose tissue proteomic analysis in ketotic or healthy Holstein cows in early lactation1. J. Anim. Sci..

[26] Shi K., Liu X., Li H., Lin X., Yan Z., Cao Q., Zhao M., Xu Z., Wang Z. (2017). Menin Modulates Mammary Epithelial Cell Numbers in Bovine Mammary Glands Through Cyclin D1. J. Mammary Gland. Biol. Neoplasia.

[27] Buitenhuis B., Janss L.L., Poulsen N.A., Larsen L.B., Larsen M.K., Sorensen P. (2014). Genome-wide association and biological pathway analysis for milk-fat composition in Danish Holstein and Danish Jersey cattle. BMC Genom..

[28] Khan M.Z., Khan A., Xiao J., Ma Y., Ma J., Gao J., Cao Z. (2020). Role of the JAK-STAT Pathway in Bovine Mastitis and Milk Production. Animals.

[29] Rainard P., Cunha P., Bougarn S., Fromageau A., Rossignol C., Gilbert F.B., Berthon P. (2013). T helper 17-associated cytokines are produced during antigen-specific inflammation in the mammary gland. PLoS ONE.

[30] Zhang K., Zhang M., Su H., Zhao F., Wang D., Zhang Y., Cao G., Zhang Y. (2024). Regulation of Inflammatory Responses of Cow Mammary Epithelial Cells through MAPK Signaling Pathways of IL-17A Cytokines. Animals.

[31] Hirosawa T., Kawamoto S., Shimizu T. (2019). SAPHO syndrome. BMJ Case Rep..

[32] Wei J.L., Villwock J.A. (2021). Balance Versus Integration: Work-Life Considerations. Otolaryngol. Clin. N. Am..

[33] Hu Z.L., Reecy J.M. (2007). Animal QTLdb: Beyond a repository. A public platform for QTL comparisons and integration with diverse types of structural genomic information. Mamm. Genome.

[34] Liu Y., Han B., Zheng W., Peng P., Yang C., Jiang G., Ma Y., Li J., Ni J., Sun D. (2023). Identification of genetic associations and functional SNPs of bovine KLF6 gene on milk production traits in Chinese holstein. BMC Genom. Data.

[35] Iqbal A., Yu H., Jiang P., Zhao Z. (2022). Deciphering the Key Regulatory Roles of KLF6 and Bta-miR-148a on Milk Fat Metabolism in Bovine Mammary Epithelial Cells. Genes.

[36] Tarcic G., Avraham R., Pines G., Amit I., Shay T., Lu Y., Zwang Y., Katz M., Ben-Chetrit N., Jacob-Hirsch J. (2012). EGR1 and the ERK-ERF axis drive mammary cell migration in response to EGF. FASEB J..

[37] Rusidze M., Adlanmerini M., Chantalat E., Raymond-Letron I., Cayre S., Arnal J.F., Deugnier M.A., Lenfant F. (2021). Estrogen receptor-alpha signaling in post-natal mammary development and breast cancers. Cell Mol. Life Sci..

[38] Li Y., Han B., Liu L., Zhao F., Liang W., Jiang J., Yang Y., Ma Z., Sun D. (2019). Genetic association of DDIT3, RPL23A, SESN2 and NR4A1 genes with milk yield and composition in dairy cattle. Anim. Genet..

[39] Chen Q., Mi S., Xing Y., An S., Chen S., Tang Y., Wang Y., Yu Y. (2024). Transcriptome analysis identifies the NR4A subfamily involved in the alleviating effect of folic acid on mastitis induced by high concentration of Staphylococcus aureus lipoteichoic acid. BMC Genom..

[40] Tribout T., Croiseau P., Lefebvre R., Barbat A., Boussaha M., Fritz S., Boichard D., Hoze C., Sanchez M.P. (2020). Confirmed effects of candidate variants for milk production, udder health, and udder morphology in dairy cattle. Genet. Sel. Evol..

[41] Berenguier C., Chen X., Allegrini B., Guizouarn H., Borgese F., Etchebest C., Soriani O., Rapetti-Mauss R. (2025). Cancer-associated loss-of-function mutations in KCNQ1 enhance Wnt/beta-catenin signalling disrupting epithelial homeostasis. Oncogene.

[42] Chen Y., Chen L., Lun A.T.L., Baldoni P.L., Smyth G.K. (2025). edgeR v4: Powerful differential analysis of sequencing data with expanded functionality and improved support for small counts and larger datasets. Nucleic Acids Res..

[43] Park Y., Wu H. (2016). Differential methylation analysis for BS-seq data under general experimental design. Bioinformatics.

